# Editorial: Weak Interactions in Molecular Machinery

**DOI:** 10.3389/fmolb.2018.00117

**Published:** 2019-01-23

**Authors:** Rivka L. Isaacson, Irene Díaz-Moreno

**Affiliations:** ^1^Department of Chemistry, King's College London, London, United Kingdom; ^2^cicCartuja, Institute for Chemical Research (IIQ), University of Seville - CSIC, Seville, Spain

**Keywords:** transient interactions, molecular machineries, structural biology, biophysics, weak contacts

Individuals are often sustained by intense relationships with others, although few would deny the importance of those more transient interactions with service providers, colleagues, and casual acquaintances in facilitating our continued existence. These latter associations are necessarily and conveniently weaker. Nobody has time for a deep conversation with everyone they encounter during a day, and society, like so many complex systems, runs on a hierarchy of interaction strengths. Similarly, inside each crowded cell of our bodies, functions arise when communication is established through biomolecules that physically and specifically contact each other in a concerted manner to transmit messages efficiently.

“Molecular sociology” within cells involves binding events between macromolecules, such as protein–protein, protein–nucleic acid, protein–carbohydrate, and protein–membrane interactions, that are acutely orchestrated to sustain the life–death balance. These interactions between biomolecules occur on a wide range of timescales. Stable complexes, with lifetimes ranging from minutes to days, involve high affinity and high specificity binding. Amongst many others, these include irreversible enzyme inhibition and the assembly of proteins supporting the cell's ultrastructure.

Weak complexes are characterized by a fine balance between specificity of binding and fast turnover rate, with the majority displaying equilibrium dissociation constants within the micromolar or even millimolar range. Perhaps counter-intuitively, these weaker molecular recognition mechanisms are not uncommon and play key roles in many biological processes, including electron transfer chains in respiration and photosynthesis and the cell signaling cascades involving kinases and phosphatases. Due to technical limitations, weak complexes remain poorly understood despite their critical role in many biological events inside the cell.

Over the last few years, specific tools have been developed to analyse more transient intermolecular interactions, including NMR paramagnetic relaxation enhancement and kinetic approaches. These methods have recently been complemented by high-throughput techniques to identify novel biomolecules that weakly bind to each other. Such advances make the analysis of the transient biointeractome (the so-called *trans-biointeractome*) more affordable. Overall, *trans-biointeractome* analysis remains highly dependent on the specific technique employed. In addition, the dynamics of the system can enhance the strength of interactions that are weak. Similarly, “one person may not be able to pull a truck from the mud but many would be able to.” This may also be the reason that interactions detected under controlled conditions *in vivo* become extremely difficult to study in isolation. Protein compartmentalization, elevated protein levels at defined moments of the cell cycle, multivalent binding, can increase (temporarily) the affinities of transient interactions, suggesting that the so-called *trans-biointeractome* is inherently plastic and that it evolves during cell lifespan. Therefore, the study of weakly interacting systems can be reliably tackled in depth only by an integrative and holistic approach combining *in vitro* strategies with other methods to detect such interactions within the cell. We are delighted by the breadth of techniques and systems covered in the articles of our research topic.

Unsurprisingly, given its well-documented strengths in measuring weak binding, NMR spectroscopy features heavily as a method used in this Special Issue in *Frontiers in Molecular Biosciences*.

Nieto provides a comprehensive mini review on using NMR to investigate binding between carbohydrates and proteins, which is particularly useful given the challenges of isotopically labeling glycans. In their original research article, the group headed by Krysztofinska et al. use NMR alongside X-ray crystallography and isothermal titration calorimetry (ITC) to analyse the structure and binding of different heat shock chaperones, via a carboxylate clamp mechanism, to a co-chaperone involved in targeting proteins to membranes. Multiple proteins competing for the same binding site, as analyzed using NMR, is a theme that continues in the research article by Adinolfi et al. who also employ small-angle X-ray scattering (SAXS), biolayer interferometry (BLI), and native mass-spectrometry (MS) to dissect the dual regulation of bacterial Iron Sulfur cluster biogenesis by two proteins, CyaY and IscX. Tossavainen et al. also use NMR and SAXS, alongside Molecular Dynamics simulations to explore the variable affinity interactions between *Staphylococcus aureus* cell-wall digester, lysostaphin, and its substrates.

Surface plasmon resonance (SPR) is another widely used technique to measure interactions, and along with microarray technology, is employed by Watson et al. to establish the efficacy of a peptide inhibitor in blocking an important weak interaction between an SH2 domain and its phosphotyrosine target. This is a step on the way to rational drug design for cancer.

The importance and unique attributes of intrinsically disordered proteins (IDPs) are becoming increasingly recognized, acknowledged and valued. Arbesú et al. feed into this theme by presenting a fascinating mini review on the intramolecular “fuzzy” interactions that occur within intrinsically disordered domains of proteins. This mini-review perfectly links with the Frontiers topic on *Function and Flexibility: Friend or Foe?*

Perhaps the most self-contained story in our collection, that truly conveys the variety of weak interactions in a well-characterized pathway, is the review from the group of Forcada-Nadal et al. on the PII-NAGK-PipX-NtcA regulatory axis of cyanobacteria, which beautifully compiles the information that led to an understanding of this network. Clathrin-mediated endocytosis is an elegant process as delineated in a review from. Smith et al. who examine the vital roles of weak interactions in this choreographed cellular system. On the other hand, it becomes more and more evident that there is a need for a transition from a static to a dynamic point of view in order to take into account the biological environment during RNA binding and RNA metabolism, as reviewed by the team of García-Mauriño et al. Actually, the understanding of the mRNAs processing in highly dynamic and often transient macromolecular complexes also remains challenging.

The subjects covered in our research topic span a wide range of biological questions and methodology. We believe this is merely the tip of the iceberg and that the future will bring vast insight into the importance of weak interactions in molecular machinery.

## Author Contributions

All authors listed have made a substantial, direct and intellectual contribution to the work, and approved it for publication.

### Conflict of Interest Statement

The authors declare that the research was conducted in the absence of any commercial or financial relationships that could be construed as a potential conflict of interest.

